# A targeted controlled force injection of genetic material *in vivo*

**DOI:** 10.1038/mtm.2016.16

**Published:** 2016-03-30

**Authors:** Gustaf Ahlén, Lars Frelin, Fredrik Holmström, Grant Smetham, Steve Augustyn, Matti Sällberg

**Affiliations:** 1 Department of Laboratory Medicine, Division of Clinical Microbiology, Karolinska Institutet, Karolinska University Hospital Huddinge, Stockholm, Sweden; 2 Team Consulting Limited, Cambridge, UK

## Abstract

A general limitation in gene delivery is the cellular uptake in lager animals including humans. Several approaches have been tested including liposomes, micro-needles, *in vivo* electro-transfer, ballistic delivery, and needle-free delivery. All these techniques have individual limitations. One approach reproducibly delivering genetic material in muscle tissue in nonhuman primates is hydrodynamic injection, a forced injection of a volume equaling the volume of the tissue to be transfected thereby causing an increased local pressure resulting in an improved uptake of genetic material. We transferred the principle of hydrodynamic injection to a device, where a small injection volume can be delivered to a targeted tissue volume, termed *in vivo* intracellular injection (IVIN). The device is based on needle(s) with apertures along the needle shafts, where multiple needles can fix the tissue volume to be transfected. The apertures direct the injection from a central needle outward or inward to the centroid of a geometric arrangement thereby targeting the tissue to be transfected. With a controlled force, this results in a targeted injection with increased transfection efficiency. We here show that the IVIN technology reproducibly improved plasmid uptake and expression and the immunogenicity. The IVIN technology can be generally applied to a targeted delivery of genetic materials.

## Introduction

DNA, and now also RNA, vaccines are relatively simple to produce and can streamline and simplify a vaccine production line. A major limitation of DNA/RNA vaccines is the poor uptake in cells of larger animals. This may be overcome by the use of various more or less advanced delivery techniques that can be extended for human use.^1–4^ In smaller animals, such as mice and smolt (young salmon or rainbow trout), the genetic material is taken up surprisingly effective.^5,6^ One reason for this is most likely the disproportionally large volume that is used for the injection, where fish weighing around 1–2 g are injected with DNA in a volume of 50 µl.^7^ Also, a 50 µl volume is commonly used to inject genetic material into the tibialis cranialis muscle of mice, which is almost the same volume as the muscle itself. Injection of the same volume in the mouse quadriceps muscle is significantly less effective. Hence, there is most likely a transiently increased hydrostatic pressure in the tissue that improves the uptake of the genetic material. A transiently increased hydrostatic pressure has in fact been applied to gene transfer and has been termed a hydrodynamic injection. This has been effectively applied to *in vivo* transfection of mouse livers^8,9^ and muscle tissue in nonhuman primates.^6^ The principle is that a tissue is overloaded by a forced injection with a volume that equals the volume of the tissue to be transfected. *E.g.*, the example of the nonhuman primate would correspond to an injection of around 100 ml into a limb. Such a volume is of course not acceptable for most future treatments where a small local uptake of the genetic material is desired, as in vaccinations. Thus, it would be of interest to apply the hydrodynamic injection technology with much smaller injection volumes in larger animals including humans. In addition, to make the use of gene therapy and genetic vaccinations more available, the device should be as simple as possible, independent of the physical capabilities of the operator, and preferentially not need any compressed gas, electromechanical drives, or advanced apparatus, such as *in vivo* electrotransfer or a ballistic delivery device such as the gene gun.

Herein, we describe the use of a new delivery device, termed *in vivo* intracellular injection (IVIN) device, which delivers a small injection volume, by the use of a compression spring, to a targeted tissue volume resulting in an improved uptake and expression of the vaccine antigen.

## Results

### Inventing the IVIN device

**Figure 1:**
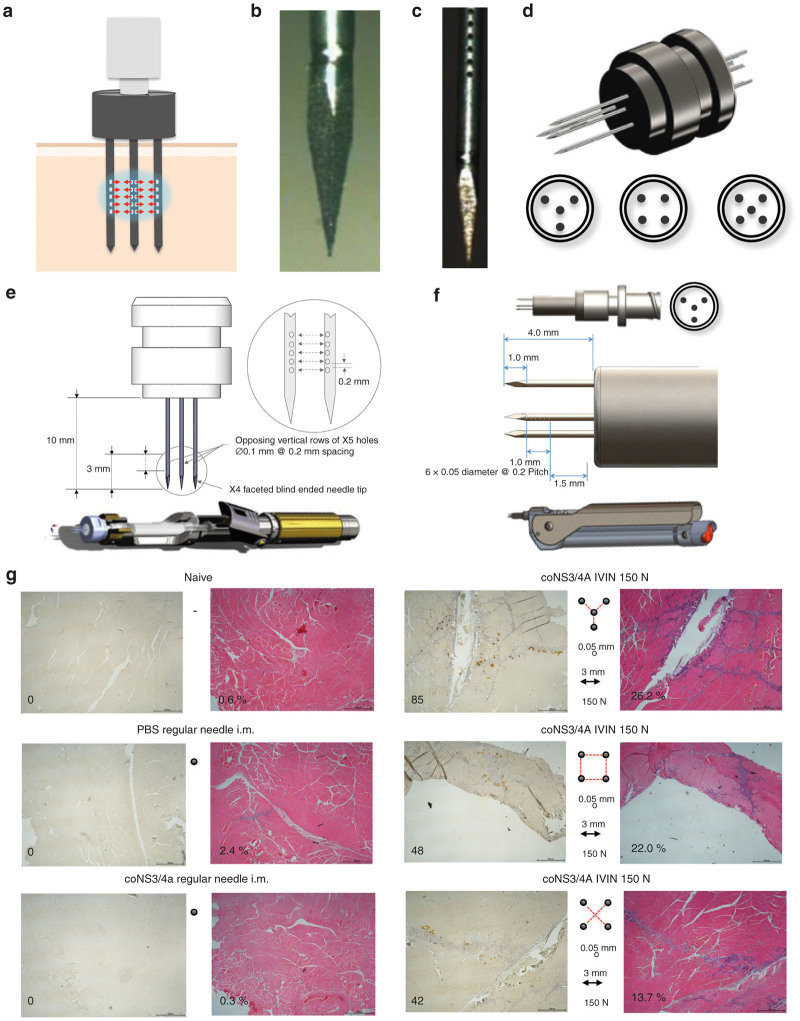
Design of the *in vivo* injection needle (IVIN) and delivery device. The concept of the IVIN technology (**a**) with all apertures facing a central and isolated area of the injected muscle tissue (**b**) with the end of the needles sealed and (**c**) apertures arranged along the needle shaft. (**d**) The different needle orientations (Y, O, and X) evaluated in rabbits is shown. (**e** and **f**) A draft of the different IVIN and delivery devices for i.m. injection in larger animals (**e**) and skin in larger animals and i.m. injection in mice (**f**), respectively. (**g**) Immunohistochemical staining of NS3-protein expression (peroxidase) and inflammation (H&E) in rabbit muscles 72 hours after injection with 0.9 mg coNS3/4A-pVAX1 using regular needle or the different IVIN needle configurations. The number of NS3-positive cells (brown) and the percentage of inflammation in each section have been indicated. H&E, hematoxylin–eosin; IVIN, *in vivo* intracellular injection.

We started designing a device that could use the advantages of uptake of genetic material mediated by the targeted controlled force injection but that could be used for injection of volumes of 1 ml or less. We envisioned that if one could isolate a tissue volume and overload the same tissue with a solution containing genetic material, this would correspond to a hydrodynamic injection. This could be achieved using one or more needles where the apertures are located along the needle shaft (Figure 1a–c). With multiple needles arranged in a circular pitch, and possibly with a central needle, so that the apertures oppose each other, an effective overloading of the tissue should be achieved. The needles located along the circle will fix the tissue that is targeted so that the tissue does not flex during the injection (Figure 1d). To avoid bending of the needles during insertion, the needles were designed with trocar tips (Figure 1b). A number of different needle configurations were explored (Figure 1d), in order to understand what impact this would have on vaccine take up. It was important that these different hub designs should be able to connect to commercially available syringes to reduce development risks and provide a more straightforward route to a commercial product. Additional factors that may affect the efficiency of the uptake of the genetic material are the spacing between the needles, the number of apertures on the shafts, the size of the apertures, and the injection force. In order to control the injection force and provide a consistent tissue-overloading effect that would not be limited by the capabilities of the device operator, we designed spring-loaded injection devices. One for i.m. delivery in larger animals such as rabbits and pigs with a larger spring and greater spacing between needles (Figure 1e) and a smaller for i.m. delivery in mice or skin delivery in larger animals (Figure 1f).

### Factors influencing transfection efficiency and inflammation *in vivo* using the IVIN device

To identify the key parameters that determine the efficiency of the IVIN technology, we first performed gene transfer experiments in rabbits, which we here consider as a larger animal as the muscle sizes better resemble those of humans. The parameters tested included the size of apertures (50 or 100 µm), the injection force (50–225 N), the distance between needles (3 or 6 mm), and the needle configuration (O-, X-, or Y-shaped pattern).

These parameters were tested with the readout being the ability to transfect rabbit tibialis anterior muscles *in vivo* and to cause a local inflammation using a plasmid expressing the hepatitis C virus nonstructural (NS) 3/4A proteins, administered as a 0.3 ml injection with 3 mg/ml of DNA. A general pattern was that both transfection and inflammation seemed to appear along the needle tracks (Figure 1g). Subgroups were grouped into larger groups to allow for statistical analysis. For example, in the two groups comparing the apertures, subgroups include different injections forces, different needle distances, and different needle configuration. The purpose of this type of comparison was to find parameters that could override the influence of the subgroups.

**Figure 2:**
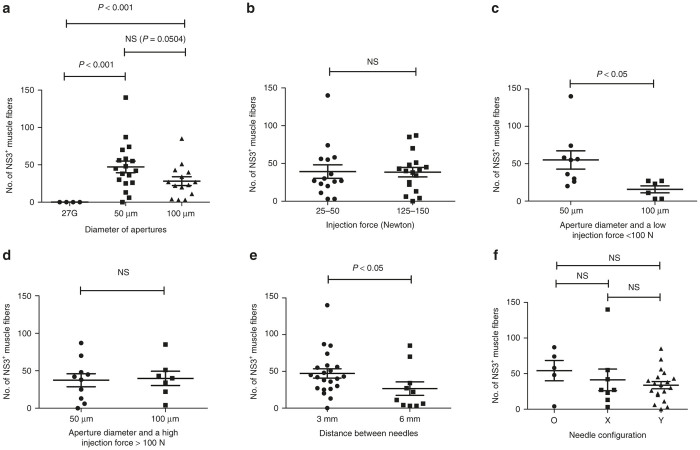
Factors influencing transfection efficiency *in vivo* using the IVIN. To identify factors influencing the transfection efficiency *in vivo* in muscles, rabbits were injected with 0.9 mg coNS3/4A-pVAX1 using several different IVIN prototypes and injection forces. (**a**) The effect on NS3 protein expression using different apertures size (50 or 100 μm) is compared to a regular 27G syringe. (**b**) Comparison of the highest (125–150 N) and the lowest (25–50 N) injection forces used by the ADU. The different aperture diameter and its effect using (**c**) low and (**d**) high injection force. Influence of the distance between the (**e**) needles and (**f**) the different needle configurations. The statistical difference was determined using Mann–Whitney *U*-test (*P* < 0.05; *P* < 0.001). NS, not significant, indicates no statistical difference. ADU, adjustable delivery unit; IVIN, *in vivo* intracellular injection.

With respect to these parameters, we first found that the size of the apertures seemed to affect *in vivo* transfection efficiency (Figure 2a). It is likely that an increased area of the apertures drastically reduces the dynamic force of the injection fluid. Simply considering the issue as a matter of mass flow, apertures of half the diameter (0.05 mm as opposed to 0.1 mm) will have one quarter of the cross-sectional area and so to deliver the same volume of vaccine in the same amount of time the vaccine will have to be travelling four times as quickly as it leaves the needle aperture. The model ignores the effect of tissue resistance and pressure drop across each of the apertures, but the fundamental approach is sound and has been supported with high-speed video analysis of the two aperture sizes at different injection pressures. This behavior is further supported by the subgroup analysis where injection at a lower force was less effective with larger apertures (Figure 2c), whereas at a higher injection force the aperture size was less important (Figure 2d). Thus, the injection force should not be too low. There is a practical upper limit to the injection force as using too high a force leads to the vaccine solution leaking behind the plunger bung in the syringe barrel.

**Figure 3:**
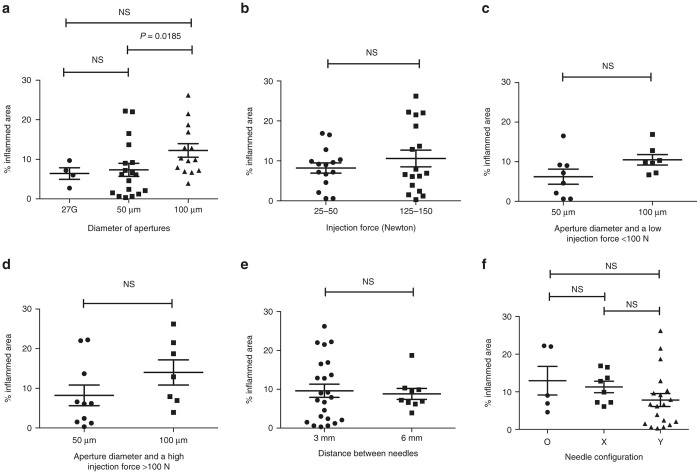
Factors influencing inflammation *in vivo* using the IVIN. Evaluation of factors influencing the inflammation associated with vaccine delivery using the IVIN needle device *in vivo* in muscles of rabbits injected with 0.9 mg coNS3/4A-pVAX1. (**a**) The effect on inflammation using different apertures size (50 or 100 μm) is compared to a regular 27G syringe. (**b**) Comparison of the highest (125–150 N) and the lowest (25–50 N) injection forces used by the ADU. The different aperture diameter and its effect using (**c**) low and (**d**) high injection force. Influence of the distance between (**e**) the needles and (**f**) the different needle configurations. The statistical difference was determined using Mann–Whitney *U*-test (*P* < 0.05). NS, not significant, indicates no statistical difference. ADU, adjustable delivery unit; IVIN, *in vivo* intracellular injection.

However, the individual role of injection force is most likely confounded by the two aperture diameters (Figure 2b). There was an influence also from the distance between the needles (Figure 2e). However, it is possible that this could be due to the fact that a larger spacing reduces the probability of two needle tracks ending up on the same histological section. A limiting factor is that the groups are too small to allow for multiple parameters at the same time. We also analyzed the influence of these parameters on the degree of local inflammation caused by the injection. The major factor that influenced the extent of inflammation at the injection site was the aperture diameter, with an increased area of inflammation seen with larger apertures (Figure 3). Thus, the size of the apertures had somewhat contradictory effects with respect to transfection and inflammation. As transfection efficiency is the key parameter, we delivered DNA using a four-needle array in a Y-shaped configuration with 50 µm apertures in following experiments (Figure 1g).

### IVIN delivery improves immunogenicity and can effectively be combined with *in vivo* electrotransfer in mice

Form the previous experiments, it was clear that the IVIN technology was superior in gene transfer in rabbits as compared to a regular needle injection. We now wanted to investigate if this is translated into an improved immunogenicity and if the performance could be further improved. We now transferred our experience from the rabbit to mice with a smaller spring-loaded hand-held IVIN device designed for mice. We tested both different injection forces and the possibility to combine the IVIN with *in vivo* electrotransfer (ET). The readouts were inflammation, transfection, and immunogenicity using different plasmids.

**Figure 4:**
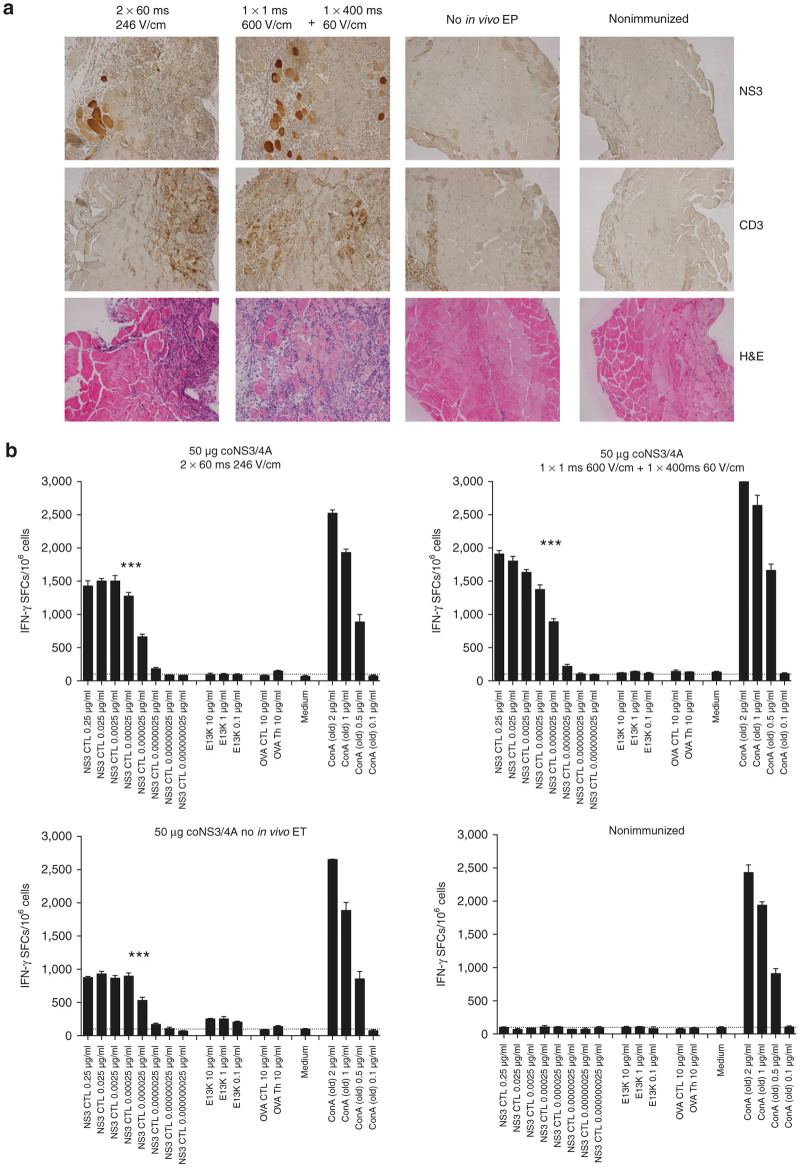
Modification of the *in vivo* electrotransfer pulse pattern. Groups of eight C57BL/6J mice were immunized once with 50 μg coNS3/4A plasmid DNA by i.m. immunization using a regular needle with or without *in vivo* ET using different pulse patterns. (**a**) Detection of HCV NS3- and CD3-expressing muscle fibers in mouse tibialis cranialis muscles 72 hours after immunization is shown in the first and second panels, respectively. Also immunohistochemical staining of inflammation (H&E) is shown in the lower panel. Two weeks after immunization, mice (five mice/group) were sacrificed and splenocytes harvested for determination of T-cell responses. (**b**) The number of interferon (IFN)-γ spot-forming cells (SFCs) by enzyme-linked immunospot (ELISpot) assay after stimulation with either a cytotoxic T lymphocyte peptide (NS3-CTL) or a Th peptide (E13K) in immunized and nonimmunized C57BL/6J mice is shown. Results are given as the mean SFCs/10^6^ (+SD) with a cutoff set at 50 SFCs/10^6^ splenocytes. The statistical difference shown indicates a group that is significantly different from all other groups (***P < 0.001, by comparing area under the curve (AUC) and analysis of variance (ANOVA)). ET, electrotransfer.

We first evaluated two different pulse patterns for the *in vivo* ET with a regular injection needle. One based on our previously used with two 60 ms pulses of 246 V/cm and the other with a short high-voltage pulse (1 ms 600 V/cm) followed by a long low-voltage pulse (400 ms 60 V/cm). The high–low pulse pattern induced a stronger T-cell response without causing increased tissue damage (Figure 4). Consistent with our previous experience, *in vivo* ET enhanced plasmid uptake, inflammation, and antigen expression (Figure 4).

**Figure 5:**
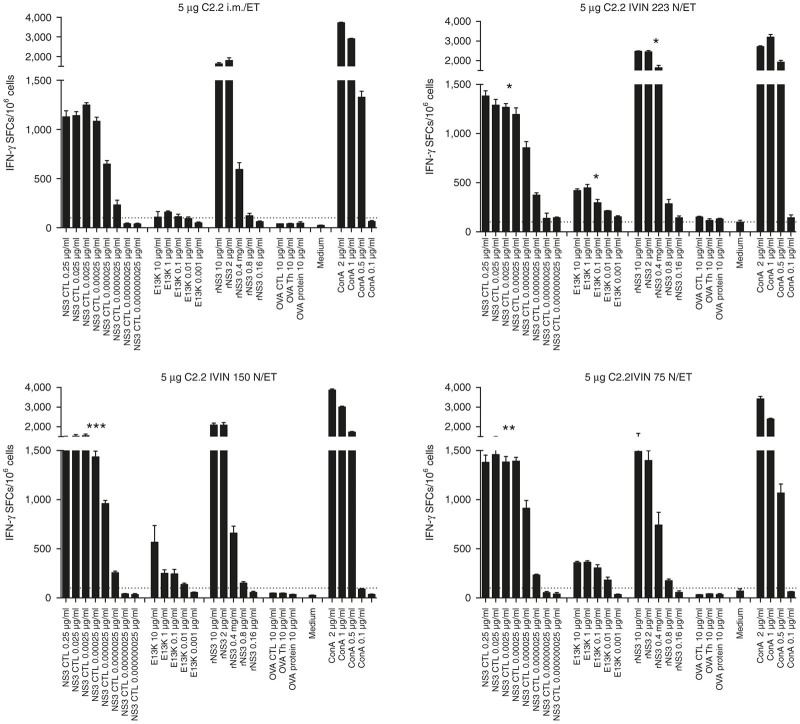
Evaluation of different injection forces for optimal immune priming of T cells. Groups of five C57BL/6J mice were immunized once with 5 μg of C2.2-pVAX1 plasmid in a volume of 30 μl by i.m. immunization using the IVIN technology and three different injection forces (75, 150, and 223 N) followed by *in vivo* ET with a pulse pattern of 1 ms 600 V/cm × 400 ms 60 V/cm. Two weeks after immunization, the mice were sacrificed and splenocytes harvested for determination of T-cell responses. A comparison of the number of IFN-γ spot-forming cells (SFCs) by enzyme-linked immunospot (ELISpot) assay after stimulation with a NS3-cytotoxic T lymphocyte peptide, a Th peptide (E13K), or a recombinant NS3 (rNS3) protein between the groups of mice immunized using different injection forces. Results are given as the mean SFCs/10^6^ (+SD) with a cutoff set at 50 SFCs/10^6^ splenocytes. The statistical differences shown indicate a group that is significantly different from the group immunized using regular 27G needle (i.m./ET) (*P < 0.05 and **P < 0.01, and ***P < 0.001, by comparing area under the curve (AUC) and analysis of variance (ANOVA)). ET, electrotransfer; IVIN, *in vivo* intracellular injection.

As most reagents for immunological characterization are available for mice, we further evaluated the IVIN in mice. We first evaluated the possibility of combining the IVIN technology using different injection forces ranging from 9 to 20 N (a 10-fold scale down from the larger device injecting a 10 times larger volume) with *in vivo* ET. This showed that IVIN could be effectively combined with *in vivo* ET (Figure 5). Also, regardless of the injection forces used, did the IVIN consistently induce stronger immune responses than a regular needle in mice when combined with *in vivo* ET (Figure 5), supporting the data from the rabbits (Figures 1–3).

**Figure 6:**
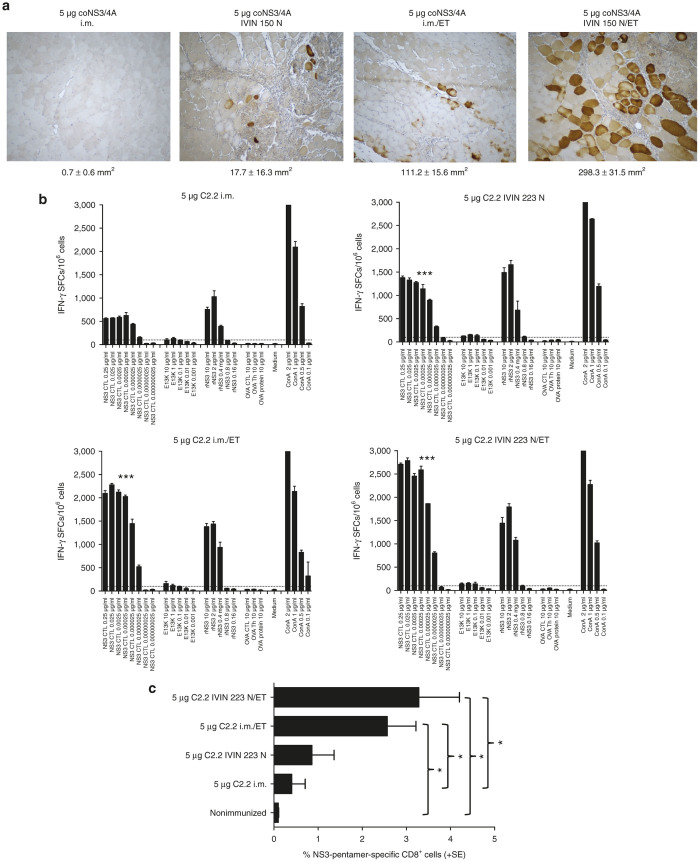
Importance of combination of IVIN and *in vivo* ET. (**a**) Detection of HCV NS3 protein-expressing muscle fibers from mouse *tibialis cranialis* muscles in groups of mice (three mice/group) 72 hours after immunization with 5 μg coNS3/4A plasmid DNA is shown. The immunizations have been done using regular needle alone or in combination with *in vivo* ET or by IVIN with or without *in vivo* ET. The number of NS3protein-expressing (peroxidase) hepatocytes/2.5 mm^2^ (mean ± SD) is given for each group of mice. (**b**) Groups of five C57BL/6J mice were immunized once with 5 μg C2.2-pVAX1 alone or in combination with 5 μg IL-12 plasmid DNA by i.m. immunization as described in **a**. Two weeks after immunization, the mice were sacrificed and splenocytes harvested for determination of T-cell responses. The number of interferon (IFN)-γ spot-forming cells (SFCs) by enzyme-linked immunospot (ELISpot) assay after stimulation with a NS3-cytotoxic T lymphocyte peptide, a Th peptide (E13K), or recombinant NS3 protein in the different groups of immunized mice is shown. Results are given as the mean SFCs/10^6^ (+SD) with a cutoff set at 50 SFCs/10^6^ splenocytes. Statistical differences have been evaluated between the different groups of immunized mice as ***P < 0.001 using area under the curve (AUC) and analysis of variance (ANOVA). (**c**) The expansion of NS3-specific CD8+ T cells was determined using direct *ex vivo* pentamer staining. GAVQNEVTL epitope-specific CD8+ T cells are shown as the mean percentage (+SE) of NS3-pentamer-positive CD8+ T cells. The statistical difference (*P < 0.05) was determined using Mann–Whitney *U*-test. ET, electrotransfer; IVIN, *in vivo* intracellular injection.

To better understand the mechanism for the improved immunogenicity in mice, we also analyzed the *in vivo* transfection efficiency determined as NS3-expression (Figure 6a). This confirmed our observations regarding the IVIN from the rabbit, with higher transfection efficiencies using the IVIN as compared to a regular needle and further improved transfection when IVIN was combined with *in vivo* ET (Figure 6a). Importantly, we could show that the *in vivo* transfection efficiency was paralleled by an increased immunogenicity determined by both the number of IFNγ-producing cells and the total number of epitope-specific cytotoxic T lymphocytes (CTLs) (Figure 6b,c). Taken together, these data clearly confirmed that the IVIN technology had a superior transfection efficiency resulting in a superior immunogenicity, as compared to a regular needle (Figures 5 and 6).

**Figure 7:**
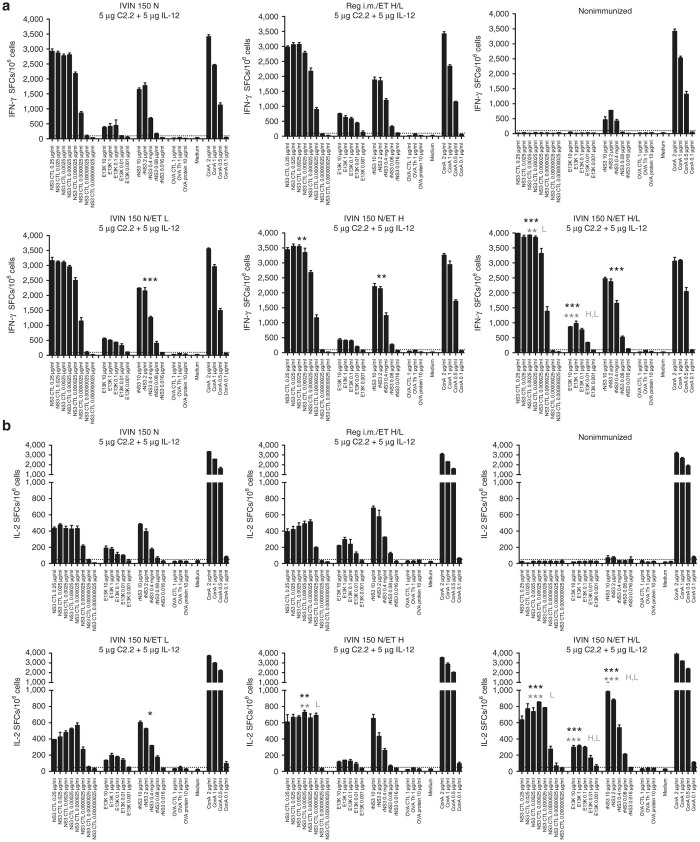
Evaluation of different *in vivo* ET pulses. Groups of five C57BL/6J mice were immunized once with 5 μg of C2.2-pVAX1 plasmid in combination with 5 μg mIL-12-pORF1 in a volume of 30 μl by i.m. immunization using the IVIN technology followed by *in vivo* ET using different pulse settings of H = 1 ms 600 V/cm and L = 400 ms 60 V/cm. Two weeks after immunization, the mice were sacrificed and splenocytes harvested for determination of T-cell responses. A comparison is done on the number of (**a**) IFN-γ and (**b**) IL-2 spot-forming cells (SFCs) by enzyme-linked immunospot (ELISpot) assay after stimulation with a NS3-cytotoxic T lymphocyte peptide, a Th peptide (E13K), or a recombinant NS3 (rNS3) protein between the groups of mice immunized using different injection forces. Results are given as the mean SFCs/10^6^ (+SD) with a cutoff set at 50 SFCs/10^6^ splenocytes. The statistical difference shown in black indicates a group that is significantly different from the group of mice using only IVIN 150 N. In gray, the statistical comparison is made between the three different groups of mice immunized using IVIN and *in vivo* ET (IVIN 150 N/L, H, or H/L). *P < 0.05, **P < 0.01, and ***P < 0.00 using comparing area under the curve (AUC) and analysis of variance (ANOVA). ET, electrotransfer; IVIN, *in vivo* intracellular injection.

We further dissected the combination of the IVIN device with a 150 N injection force, with different *in vivo* ET pulses to deliver a combination of a vaccine plasmid and an adjuvant plasmid-expressing IL-12 (Figure 7). The combination of the high and low pulse was superior to either pulse alone in priming HCV-specific T cells (Figure 7). In particular, the responses to the T-helper epitope (E13K; Figure 7a,b) and the priming of IL-2-producing T cells were greatly improved by the combination of the IVIN and *in vivo* ET (Figure 7b). Thus, this confirms that the combination of the IVIN and *in vivo* ET not only improves transfection efficiencies but also the immunogenicity.

### Immunogenicity of the IVIN combined with *in vivo* ET in pigs

**Figure 8:**
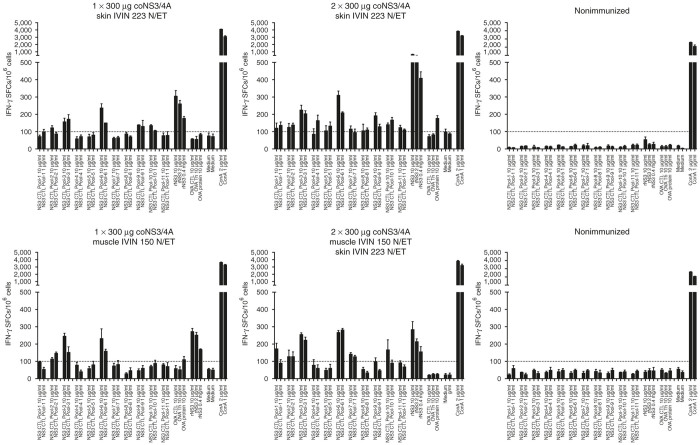
Immune responses in pigs. Six landrace pigs were either immunized one (day 0) or two (day 14) times by i.m. or skin route using 300 μg coNS3/4A-pVAX1 plasmid DNA or left untreated. Two weeks after first and second immunization, the pigs were bled for determination of T-cell responses. The number of interferon (IFN)-γ spot-forming cells (SFCs) by enzyme-linked immunospot (ELISpot) assay was determined after a 36-hour stimulation with either peptide pools covering NS3 (pool 1–11) or recombinant NS3 protein. Results are given as the mean SFCs/10^6^ (+SD) with a cutoff set at 100 SFCs/10^6^ peripheral blood mononuclear cells.

As a final test of the IVIN technology, we wanted to evaluate immunogenicity in a larger animal closer to the size of a human. We here used pigs, which are large (10–25 kg) and for which there is a reliable IFNγ-Elispot assays as readout of immunogenicity. In addition, pig skin is somewhat similar to human skin and thus suited to also test the IVIN technology for skin delivery. We used the large IVIN for i.m. delivery and the small IVIN for skin delivery. A total of four pigs were immunized with different combinations of the coNS3/4A vaccine. We found that already a single injection effectively primed detectable NS3-specific T cells in pigs using both i.m. or skin delivery (Figure 8). These responses were improved in a skin-prime and skin-boost regimen (Figure 8) and also to some extent in a i.m.-prime and skin-boost regimen. Although the number of pigs was low, this shows that the combination of the IVIN and *in vivo* ET effectively primes immune responses also in larger animals. It is important to note that the vaccinations were given as a two-step procedure, with a first injection of the DNA using the IVIN followed by the ET using a separate probe manually inserted at the injection site. If this is combined into a single device providing both the targeted controlled force injection and the *in vivo* ET, the results may be further improved.

## Discussion

The major limitation with DNA and RNA vaccines is the insufficient uptake of the genetic material into the cells of larger animals and humans. It is well known that plasmid DNA is poorly taken up when injected into humans using a standard hypodermic needle due to the injection of a small volume in a large muscle tissue.^10^ The DNA will stay in the extracellular space and become degraded or excreted. Several devices have been developed with the aim at solving this problem. However, even if improvements have been made, so far no delivery devices have shown to be simple enough to enable effective human DNA/RNA vaccines. Contrary, in a 20 g mouse, the injected volume often equals the size of the muscle, whereby an overloading of the tissue causes a hydrostatic pressure in the tissue forcing the muscle cells to take up the DNA. Along the same lines, it has been shown that in large animals, such as monkeys, the isolation of muscles in the underarm by a tourniquet followed by overloading the tissue by an injection of 100 ml of plasmid-containing solution results in DNA uptake.^10^ Therefore, it would be of interest if one could isolate a smaller area of the muscle and overload it to cause the hydrostatic pressure only in that region. The aim of this study was to develop a simple injection device for a targeted controlled force delivery of genetic material into an isolated area of muscle or skin. To achieve such conditions in a large animal using a small injection volume, we aligned multiple needles on a circular pitch with apertures laser cut on the side of the shafts and with sharp trocar ends. In this way, the solution is injected centrally into the muscle volume locked by the surrounding needles. Hereby, we effectively isolate a given volume of tissue that will become overloaded with the injected vaccine to promote a localized uptake of genetic material. Initial experiments in rabbits supported the theory in practice resulting in an improved antigen expression *in vivo*. However, through mathematical modeling, we realized the importance of controlling the injection speed. We therefore developed a spring-loaded delivery device that delivers the injection at a controlled force. Next, we evaluated key parameters such as size of apertures, the injection force, the distance between needles, and the needle configuration that determine the transfection efficiency and the inflammation caused by the IVIN technology. The size of the apertures affected *in vivo* transfection efficacy since smaller (50 μm) apertures seemed to have a higher transfection efficacy than the larger (100 μm) apertures. However, at higher injection forces (<100 N in the larger device), no significant difference was observed between the different aperture sizes with respect to transfection efficacy or inflammation. Based on the overall results, we decided to deliver DNA using a four-needle array in a Y-shaped configuration with 50 µm apertures. To perform more detailed studies on the IVIN technology, we transferred our experience from the rabbits to mice using a smaller spring-loaded hand-held IVIN specially designed for mice. This allowed us to evaluate not only transfection and inflammation but also more importantly the effect on immunogenicity. Furthermore, we could now test the role of different injection forces and the possibility to combine IVIN with *in vivo* ET. We have previously shown that *in vivo* ET greatly improves transfection efficacy and immunogenicity of DNA vaccines.^11–13^ We first evaluated whether different pulse pattern for the *in vivo* ET had an impact on the transfection efficacy and immune activation. A high–low pulse combination pattern induced a high transfection rate, influx of CD3+ cells, and a strong T-cell response. This may be explained by the fact that the higher pulse increases the permeability of the cell membranes, and that the lower pulse promotes electrophoretic transfer of the plasmids inside the cells.^14^ Next, we evaluated the combination of IVIN technology and *in vivo* ET. The IVIN was consistently better at inducing immune reposes as compared to a regular needle. This suggested to us that the addition of *in vivo* ET might even further improve the performance of the IVIN. To test the importance of both IVIN and *in vivo* ET, we determined the HCV NS3-protein expression after injection with and without IVIN and *in vivo* ET. This showed that both *in vivo* ET and IVIN had effects on the transfection efficacy when used alone; however, the combination of these was superior. We further tested the combination of the IVIN device with a 150 N injection force with different *in vivo* ET pulses to deliver a combination of plasmids. The combination of a high and a low pulse was superior to either pulse alone in priming HCV-specific T cells reaching IFN-γ responses up to 4,000 spot-forming cells (SFCs)/10^6^ after a single immunization of only 5 μg vaccine. This is consistent with previous observations where different pulses were combined.^14^ Thus, the combination of IVIN and *in vivo* ET was highly effective in mice and should therefore be further explored in larger animals. As a first set of experiments, we used pigs since they resemble humans in size of muscle tissue and share similarities with human skin. Using a two-step administration procedure, with a first IVIN injection followed by *in vivo* ET, we found that both i.m. and skin immunization primed HCV-specific IFN-γ responses supporting that the combined IVIN technology works in larger animals. However, the major advantage of the IVIN technology will be when the injection needles also acts a electrodes for the ET, since then the whole procedure is done in a single step. Such a device is currently under development.

We here describe a new injection device, the IVIN, which can achieve a targeted controlled force injection with improved uptake of genetic material in a targeted small volume of tissue *in vivo*. The IVIN technology can effectively be used in combination with *in vivo* ET, which further improves transfection and immunogenicity. This gene transfer technology that can be applied on any DNA or RNA vaccine is a promising step in making these types of vaccine a reality in humans.

## Materials and Methods

### Animals

Inbred female C57BL/6J (H-2^b^) mice were housed at Karolinska Institutet, Division of Comparative Medicine, Clinical Research Center, Karolinska University Hospital, Huddinge, Sweden. The mice were purchased from approved vendors (Charles River Laboratories, Sulzfeld, Germany and the Jackson Laboratory, Bar Harbor, ME). The mice were caged at 5–10 mice per cage and fed with a commercial diet (RM3 (p) PL IRR diet; Special Diet Service) with free access to food and water. All mice were 6–10 weeks of age at the start of the experiment. New Zealand White (NZW) rabbits weighing 2.5–3.5 kg, and landrace pigs of 6 weeks of age, were purchased from commercial vendors and were kept at Adlego Biomedical AB, Astrid Fagræus Laboratory, Karolinska Institutet, Solna, Sweden. All animal studies were approved by the Ethical Committee for Animal Research.

### Plasmid DNA

The codon-optimized (co) NS3/4A-pVAX1 genotype 1a plasmid (GenBank accession number: AR820945.1; http://www.nbci.nlm.nih.gov/genbank) has been described previously.^15^ In addition, a codon-optimized fusion construct of NS3/4A and stork HBcAg (coStork-HBcAg) C2.2 has been described previously (Levander et al., manuscript submitted). The C2.2 (GenBank accession number: GZ869906.1) contain an NS3-NS4A cleavage site between NS3 and NS4A and an NS4A-NS4B cleavage site between NS4A and stork-HBcAg. NS3, NS4A, and stork-HBcAg are separated by NS3/4A proteolytic cleavage, and the coStork-HBcAg contain modifications consisting of the introduction of three NS3-NS4A cleavage sites in the coStork-HBcAg gene sequence, allowing the expressed NS3/4A protease to cleave the modified Stork-HBcAg into four parts (Stork-HBcAg: aa 1–66, aa 67–132, aa 133–198, and aa 199–264). Mouse IL-12 (pORF-mIL-12) was purchased from InvivoGen (San Diego, CA). Plasmids were grown in competent TOP10 *E. coli* (Life Technologies, Carlsbad, CA) and purified using Qiagen EndoFree Plasmid Purification Kit according to the manufacturer’s instructions (Qiagen, Hilden, Germany). Plasmid DNA concentration was determined spectrophotometrically, and the purified DNA was dissolved in sterile phosphate-buffered saline at concentrations of 2 mg/ml. Restriction enzyme digest and sequencing (Eurofins MWG Operon, Ebersberg, Germany) was performed to control the size of the insert and to ensure correct nucleotide sequence. Plasmid constructs were analyzed for protein expression and proteolytic activity by an *in vitro* transcription and translation assay (Promega, Madison, WI) and by transient transfection and western blot analysis as described elsewhere (Levander et al., manuscript submitted).^15,16^

### Peptides and proteins

To study HCV CD4^+^ and CD8^+^ T-cell responses, we used previously identified H-2^b^-restricted CTL and T helper epitopes: HCV NS3-CTL peptide (H2-D^b^ aa sequence: GAVQNEVTL (NS3 aa 1629–1637, gt1a)),^17^ HCV NS3-Th peptide (aa sequence: EIPFYGKAIPLEAIK (E13K aa 1372–1386, gt1a)).^18^ As control peptides, we used ovalbumin: SIINFEKL (OVA 257–264, CTL-epitope) and ISQAVHAAHAEINEAGR (OVA 323–339, Th-epitope) synthesized by automated peptide synthesis as described previously (ChronTech Pharma AB, Huddinge, Sweden).^19^ Recombinant HCV NS3 protein gt1a (aa 1207–1612) were produced in *E. coli* and purified as described.^20^ Chicken egg albumin (OVA) and Concanavalin A (ConA) were purchased from Sigma Aldrich (St Louis, MO).

### IVIN device for delivery of DNA vaccine

The IVIN device is a multineedle injection device where the sharp end of the needles has been sealed and several (12–36) new 0.1- or 0.05-mm diameter apertures have been laser cut along the shaft of each needle. The IVIN needles used in this study consists of 4–5 needles arranged in an O, X, or Y (Figure 1d) shaped formation. The holes were arranged to either project toward a central point or straight to the closest neighboring needle. For rabbit and pig studies, the needles were made with 27G (O/D 0.413 mm) surgical tubing, compliant with ISO 9626. Once the holes were cut, a short length of 27G solid rod was laser welded to the end of the tube (to close the distal end), and a three facet trocar was cut into this rod to assist in penetrating the skin without causing the needle to deflect, upsetting the alignment between the needles. The needles were then welded into stainless steel hubs, and the joints were tested to confirm that there were no leaks from the joints or welds. Each needle was inspected with a microscope and subject to high-speed video analysis to ensure a good flow could be achieved through each of the apertures. The hub was then built into an assembly with a threaded Luer Lock fitting to allow it to be mounted to a standard Luer Lock syringe. It was necessary to use a Luer Lock (rather than a Luer slip) due to the high pressure generated in the delivery system. The hub was made by XL Precision Technologies (Stockton on Tees, UK). XL Precision also provided the needles for the mouse studies; these needles were made in the same manner as the larger needles but were manufactured in 30G (O/D 0.311 mm) surgical tubing. The smaller 30G needles with their thinner section presented significant challenges for laser welding. The injection is a forced injection of 30–300 μl plasmid DNA solution. To evaluate different injection forces and to standardize the injection, a spring-loaded adjustable delivery unit has been developed for both larger animals and mice. Each of the adjustable delivery unit were designed in 3D using SolidWorks CAD software and were manufactured from machined stainless steel with sliding components manufactured in brass. The injection force was adjusted by modifying the compression on a coil spring; the force was verified using a tensile testing system and then clearly marked on each of the devices. The adjustable delivery units were manufactured by Gemini Prototyping (Worminghall, UK). The systems were designed as an investigative platform and so had a lot of flexibility to adjust loads and travel in order to establish an optimum delivery profile. To determine the most effective conditions for delivery of genetic material into muscles, the following parameters were evaluated *in vitro* and by injection in rabbit muscles: hole size (0.1, 0.05 mm), distance between needles 3 or 6 mm, configuration of the needles (O, X, or Y), number of apertures (48–144), and the injection force (50–150 N) (Figure 1a–f). The complete IVIN device was designed and assembled by Team Consulting (Cambridge, UK).

### Immunization protocol

Rabbits (NZW) were immunized i.m. into the tibialis anterior muscles once with 0.9 mg DNA in a volume of 300 µl using a regular needle or the IVIN device. ET using the Cliniporator^2^ device (IGEA, Carpi, Italy) of the injected area was applied immediately after the delivery of the DNA. Three days after DNA injection, the rabbits were sacrificed, and the injected muscle was collected for immunohistochemical analysis.

In landrace pigs, both i.m. and skin delivery was evaluated in combination with ET using the Cliniporator^2^ device. A volume of 30 µl was used for the IVIN skin injections. The holes are located at a depth of 2–3 mm, thus targeting both the epidermis and the dermis. A total of six immunizations (0.5 mg/injection) on the spine of the pigs were performed with 1 cm distance between injections. The skin was thereafter electrotransferred by 0.1 ms 1,000 V/cm + 200 ms 100 V/cm (1,000 ms between pulses). Intramuscular immunizations were performed using 0.3 mg DNA in 0.3 ml solution followed by an ET protocol of 0.1 ms 600 V/cm + 200 ms 60 V/cm (1,000 ms between pulses). The pigs were boosted 2 weeks after the initial immunization, and immune responses were determined by enzyme-linked immunospot (ELISpot) assay using peripheral blood mononuclear cells isolated 2 weeks after the first and second immunization.

Groups of 5–10 (mice/group) of female C57BL/6J (wt) mice were immunized i.m. in the tibialis cranialis muscle once with doses of 50, 20, or 5 μg plasmid DNA (*e.g.*, coNS3/4A-pVAX1 or C2.2-pVAX1-pVAX1) alone or in combination with 5 μg mIL-12-pORF1^12^ in a volume of 30 μl using the IVIN or regular needle injection. Immediately following administration of the plasmid DNA, tibialis cranialis muscles were subsequently electrotransferred using the Cliniporator^2^ device (IGEA) with two 60 ms 246 V/cm pulses or 1 ms 600 V/cm pulse followed by a 400 ms 60 V/cm pulse pattern. For mice, the analysis of HCV NS3/4A-specific immune responses was performed at 2 weeks after last immunization. Determination of HCV NS3- or luciferase protein-expressing liver cells *in vivo* was performed in groups of immunized and nonimmunized mice. Presence of HCV NS3 protein expression was determined 72 hours after the targeted controlled force injection by western blot analysis.^8,21^

### Immunohistological detection of NS3, CD3, and hematoxylin–eosin in mouse and rabbit muscles

Staining was performed essentially as described previously.^8,22^ Muscle specimens were fixed in 10% phosphate-buffered formalin and subsequently embedded in paraffin. For histopathological evaluation, deparaffinized sections from the injected muscle were stained with hematoxylin–eosin or with anti-NS3 or CD3 antibody. To retrieve antigens, sections were boiled in a pressure cooker containing an antigen-unmasking solution with pH 10.0 (for CD3) or 0.01 mol/l citrate buffer (for NS3). For detection of CD3 in muscle tissue, a polyclonal rabbit antihuman CD3 antibody (Dako, Glostrup, Denmark) was used at a dilution of 1:1,000 in 0.01 mol/l citrate buffer complemented with 2.5% normal horse serum. Following incubation with the primary antibody, an ImmPRESS antirabbit Ig peroxidase detection kit was used (Vector Laboratories, Burlingame, CA) before visualization using the DAB peroxidase substrate kit (Dako) as a chromogen. For detection of NS3 protein in muscle tissue, an anti-NS3 antibody was used at a dilution of 1:1,500 (for mouse, a rabbit anti-NS3 antibody produced in-house was used) and 1:50 (for rabbit, a mouse monoclonal anti-NS3 antibody was used, Novocastra, Leica Biosystems, Nussloch, Germany). The NS3 antibodies were diluted in 0.01 mol/l citrate buffer complemented with 2.5% normal horse serum. Following incubation with the primary antibody, an ImmPRESS antimouse Ig peroxidase detection kit was used (Vector Laboratories) before visualization using the DAB peroxidase substrate kit (Dako) as a chromogen. Sections were counterstained with hematoxylin. The hematoxylin–eosin staining was performed using standard techniques described elsewhere.

### Detection of IFN-γ- and IL-2-producing T cells by ELISpot assay

Splenocytes from groups of mice (5–10 mice/group) were pooled, and peripheral blood mononuclear cell from individual pigs were tested for the presence of NS3/4A-specific T cells. We assessed the ability of NS3/4A-specific CTLs and Th cells to produce IFN-γ (mice and pigs) and IL-2 (mice) after exposure to different peptides and proteins: NS3-CTL gt1a (GAVQNEVTL), NS3-Th E13K gt1a (EIPFYGKAIPLEAIK), and recombinant HCV NS3 protein (gt1a). The following non-HCV peptides and proteins were included as control antigens: OVA-CTL (SIINFEKL), OVA-Th (ISQAVHAAHAEINEAGR), and OVA protein (OVA grade VII). Concanavalin A was used as a positive control, and medium alone was used as a negative control. Production of IFN-γ and IL-2 cytokines was determined by a commercially available ELISpot assay (Mabtech, Nacka Strand, Sweden) as described previously.^23^ The number of spots was counted using the AID iSpot reader and software version 7.0 (AID, Strassberg, Germany). The number of spots (cytokine-producing cells) was determined at each concentration of peptide or protein, and the results given as the number of IFN-γ or IL-2-producing cells per 10^6^ cells. A mean number of cytokine-producing cells of 50 (mice) and 100 (pigs) per 10^6^ cells was considered as negative.

### Quantification of HCV NS3-specific CD8^+^ T cells by pentamer staining

The frequency of NS3-specific CD8^+^ T cells was analyzed by direct *ex vivo* staining of splenocytes using the NS3 GAVQNEVTL (H2-D^b^) Pro5 pentamers (ProImmune, Oxford, UK) performed exactly as described previously.^23,24^ The following antibodies were used: antimouse CD16/32 “Fc block,” antimouse CD19-APC “clone 1D3” (BD Biosciences, San Jose, CA), and antimouse CD8-FITC “clone KT15” (ProImmune). A total of 150,000 events from each sample were acquired on a FACSCalibur or FACSVerse flow cytometer (BD Biosciences) and analyzed using the FlowJo V10.0.7 software (Tree Star, Ashland, OR).

### Statistical analysis

All comparisons were performed using GraphPad InStat 3, Macintosh (version 3.0b, 2003; GraphPad Software, San Diego, CA) and Microsoft Excel 2011, Macintosh (version 14.3.9; Microsoft, Redmond, WA). Kinetic measurements were compared using the area under the curve (AUC, Excel). Parametrical data were compared using the analysis of variance and nonparametrical data with Mann–Whitney *U*-test. Frequencies were compared using Fisher’s exact test.

## Notes

M.S. and L.F. are named inventors on patent/patents pending for the IVIN and HCV therapeutics. G.S. and S.A. are employed by Team Consulting, Cambridge, UK. G.A. and F.H. report no potential conflict of interest.

The first two authors contributed equally to this work.

## References

[bib1] Bagarazzi, ML, Yan, J, Morrow, MP, Shen, X, Parker, RL, Lee, JC et al. (2012). Immunotherapy against HPV16/18 generates potent TH1 and cytotoxic cellular immune responses. Sci Transl Med 4: 155ra138. 10.1126/scitranslmed.3004414 PMC4317299 23052295

[bib2] Ledgerwood, JE, Wei, CJ, Hu, Z, Gordon, IJ, Enama, ME, Hendel, CS et al. VRC 306 Study Team. (2011). DNA priming and influenza vaccine immunogenicity: two phase 1 open label randomised clinical trials. Lancet Infect Dis 11: 916–924. 21975270 10.1016/S1473-3099(11)70240-7 PMC7185472

[bib3] Mathiesen, I (1999). Electropermeabilization of skeletal muscle enhances gene transfer in vivo. Gene Ther 6: 508–514. 10476210 10.1038/sj.gt.3300847

[bib4] Weiland, O, Ahlén, G, Diepolder, H, Jung, MC, Levander, S, Fons, M et al. (2013). Therapeutic DNA vaccination using *in vivo* electroporation followed by standard of care therapy in patients with genotype 1 chronic hepatitis C. Mol Ther 21: 1796–1805. 23752314 10.1038/mt.2013.119 PMC3776630

[bib5] Evensen, Ø and Leong, JA (2013). DNA vaccines against viral diseases of farmed fish. Fish Shellfish Immunol 35: 1751–1758. 24184267 10.1016/j.fsi.2013.10.021

[bib6] Wolff, JA, Malone, RW, Williams, P, Chong, W, Acsadi, G, Jani, A et al. (1990). Direct gene transfer into mouse muscle *in vivo*. Science 247: 1465–1468. 1690918 10.1126/science.1690918

[bib7] Corbeil, S, LaPatra, SE, Anderson, ED and Kurath, G (2000). Nanogram quantities of a DNA vaccine protect rainbow trout fry against heterologous strains of infectious hematopoietic necrosis virus. Vaccine 18: 2817–2824. 10812224 10.1016/s0264-410x(00)00078-5

[bib8] Ahlen, G, Nystrom, J, Pult, I, Frelin, L, Hultgren, C and Sallberg, M (2005). *In vivo* clearance of hepatitis C virus nonstructural 3/4A-expressing hepatocytes by DNA vaccine-primed cytotoxic T lymphocytes. J Infect Dis 192: 2112–2116. 16288375 10.1086/498218

[bib9] Zhang, G, Budker, V and Wolff, JA (1999). High levels of foreign gene expression in hepatocytes after tail vein injections of naked plasmid DNA. Hum Gene Ther 10: 1735–1737. 10428218 10.1089/10430349950017734

[bib10] Hegge, JO, Wooddell, CI, Zhang, G, Hagstrom, JE, Braun, S, Huss, T et al. (2010). Evaluation of hydrodynamic limb vein injections in nonhuman primates. Hum Gene Ther 21: 829–842. 20163248 10.1089/hum.2009.172 PMC2938361

[bib11] Ahlén, G, Söderholm, J, Tjelle, T, Kjeken, R, Frelin, L, Höglund, U et al. (2007). *In vivo* electroporation enhances the immunogenicity of hepatitis C virus nonstructural 3/4A DNA by increased local DNA uptake, protein expression, inflammation, and infiltration of CD3+ T cells. J Immunol 179: 4741–4753. 17878373 10.4049/jimmunol.179.7.4741

[bib12] Brass, A, Frelin, L, Milich, DR, Sällberg, M and Ahlén, G (2015). Functional aspects of intrahepatic hepatitis B virus-specific T cells induced by therapeutic DNA vaccination. Mol Ther 23: 578–590. 25492563 10.1038/mt.2014.233 PMC4351461

[bib13] Nyström, J, Chen, A, Frelin, L, Ahlén, G, Koh, S, Brass, A et al. (2010). Improving on the ability of endogenous hepatitis B core antigen to prime cytotoxic T lymphocytes. J Infect Dis 201: 1867–1879. 20446851 10.1086/652808

[bib14] André, FM, Gehl, J, Sersa, G, Préat, V, Hojman, P, Eriksen, J et al. (2008). Efficiency of high- and low-voltage pulse combinations for gene electrotransfer in muscle, liver, tumor, and skin. Hum Gene Ther 19: 1261–1271. 19866490 10.1089/hum.2008.060

[bib15] Frelin, L, Ahlén, G, Alheim, M, Weiland, O, Barnfield, C, Liljeström, P et al. (2004). Codon optimization and mRNA amplification effectively enhances the immunogenicity of the hepatitis C virus nonstructural 3/4A gene. Gene Ther 11: 522–533. 14999224 10.1038/sj.gt.3302184

[bib16] Söderholm, J, Ahlén, G, Kaul, A, Frelin, L, Alheim, M, Barnfield, C et al. (2006). Relation between viral fitness and immune escape within the hepatitis C virus protease. Gut 55: 266–274. 16105887 10.1136/gut.2005.072231 PMC1856491

[bib17] Frelin, L, Alheim, M, Chen, A, Söderholm, J, Rozell, B, Barnfield, C et al. (2003). Low dose and gene gun immunization with a hepatitis C virus nonstructural (NS) 3 DNA-based vaccine containing NS4A inhibit NS3/4A-expressing tumors *in vivo*. Gene Ther 10: 686–699. 12692597 10.1038/sj.gt.3301933

[bib18] Fournillier, A, Frelin, L, Jacquier, E, Ahlén, G, Brass, A, Gerossier, E et al. (2013). A heterologous prime/boost vaccination strategy enhances the immunogenicity of therapeutic vaccines for hepatitis C virus. J Infect Dis 208: 1008–1019. 23776192 10.1093/infdis/jit267 PMC3749006

[bib19] Sällberg, M, Rudén, U, Magnius, LO, Norrby, E and Wahren, B (1991). Rapid “tea-bag” peptide synthesis using 9-fluorenylmethoxycarbonyl (Fmoc) protected amino acids applied for antigenic mapping of viral proteins. Immunol Lett 30: 59–68. 1720419 10.1016/0165-2478(91)90090-w

[bib20] Jin, L and Peterson, DL (1995). Expression, isolation, and characterization of the hepatitis C virus ATPase/RNA helicase. Arch Biochem Biophys 323: 47–53. 7487072 10.1006/abbi.1995.0008

[bib21] Ahlén, G, Derk, E, Weiland, M, Jiao, J, Rahbin, N, Aleman, S et al. (2009). Cleavage of the IPS-1/Cardif/MAVS/VISA does not inhibit T cell-mediated elimination of hepatitis C virus non-structural 3/4A-expressing hepatocytes. Gut 58: 560–569. 18689426 10.1136/gut.2007.147264 PMC2648966

[bib22] Ahlén, G, Sällberg, M and Frelin, L (2013). Methods for monitoring gene gun-induced HBV- and HCV-specific immune responses in mouse models. Methods Mol Biol 940: 239–267. 23104348 10.1007/978-1-62703-110-3_20

[bib23] Ahlén, G, Holmström, F, Gibbs, A, Alheim, M and Frelin, L (2014). Long-term functional duration of immune responses to HCV NS3/4A induced by DNA vaccination. Gene Ther 21: 739–750. 24871581 10.1038/gt.2014.48 PMC4126484

[bib24] Chen, A, Ahlén, G, Brenndörfer, ED, Brass, A, Holmström, F, Chen, M et al. (2011). Heterologous T cells can help restore function in dysfunctional hepatitis C virus nonstructural 3/4A-specific T cells during therapeutic vaccination. J Immunol 186: 5107–5118. 21430225 10.4049/jimmunol.1001790

